# Omphalocele: clinical and epidemiological profile of patients born in a tertiary care center in Rio de Janeiro

**DOI:** 10.1186/s12884-023-05741-z

**Published:** 2023-06-07

**Authors:** Matheus Sarabion Vilela Pereira, Daniela Koeller Rodrigues Vieira, Maria de Fátima M. P. Leite, Maria Auxiliadora Monteiro Villar, Carla Verona Barreto Farias

**Affiliations:** National Institute of Women, Children and Adolescents Health Fernandes Figueira/Oswaldo Cruz Foundation (IFF/FIOCRUZ), Rio de Janeiro, Brazil

**Keywords:** “Omphalocele”, “Congenital heart defects”, “Epidemiology”

## Abstract

**Background:**

The objective of this paper is to describe the clinical and epidemiological profile and the early outcomes of patients with omphalocele born in a fetal medicine, pediatric surgery, and genetics reference hospital in Rio de Janeiro - Brazil. To determine its prevalence, describe the presence of genetic syndromes, and congenital malformations, emphasizing the characteristics of congenital heart diseases and their most common types.

**Methods:**

Using Latin-American Collaborative Study of Congenital Malformations (ECLAMC) database and records review, a retrospective cross-sectional study was performed, including all patients born with omphalocele between January 1st, 2016, and December 31st, 2019.

**Results:**

During the period of the study, our unity registered 4,260 births, 4,064 were live births and 196 stillbirths. There were 737 diagnoses of any congenital malformation, among them 38 cases of omphalocele, 27 were live born, but one was excluded for missing data. 62.2% were male, 62.2% of the women were multiparous and 51.3% of the babies were preterm. There was an associated malformation in 89.1% of the cases. Heart disease was the most common (45.9%) of which tetralogy of Fallot was the most frequent (23.5%). Mortality rate was 61.5%.

**Conclusions:**

Our data showed a good correspondence with the existing literature. Most patients with omphalocele had other malformations, especially congenital heart disease. No pregnancy was interrupted. The presence of concurrent defects showed a huge impact on prognosis, since, even if most survived birth, few remained alive and received hospital discharge. Based on these data, fetal medicine and neonatal teams must be able to adjust parents counseling about fetal and neonatal risks, especially when other congenital diseases are present.

## Summary of findings

Most patients with omphalocele had other malformations, especially congenital heart disease. The presence of concurrent defects showed a huge impact on prognosis. A clear perinatal plan should be developed for couples with a multidisciplinary team, aimed at minimizing maternal morbidity and providing better care for the newborn.

## Background

Omphalocele is a congenital malformation resulting from a defect in the closure of the ventral body wall. Its exact etiology is not well elucidated, but it seems that the genesis of the defect takes place between the 6th and the 10th gestational week. During embryonic development, the midgut physiologically herniates through the umbilical cord. In omphalocele, there is a failure of the midgut to rotate and go back to the abdominal cavity, a process that normally occurs until the 12th week of gestation [1]. Its prevalence is about 1.0–2.6/10,000 live births, lower rates occur in countries where termination of pregnancy is allowed [2, 3]. Omphalocele is more common in male gender, extreme ages (young or advanced maternal age). The association of omphalocele with both multiparity and nulliparity have been described in literature, as with preterm birth, maternal obesity and/or maternal diabetes [1, 4–7].

Although it can be an isolated event, the presence of associated defects ranges widely, from 8 to 78% [8]. Heart, genitourinary and gastrointestinal tracts, and central nervous system are the most frequently involved. Septal defects, patent ductus arteriosus (PDA) and tetralogy of Fallot (TOF) are the most common cardiac malformations. But complete and uncomplete pentalogy of Cantrell is often described [9]. There is a usual association with genetic and chromosomal diseases, particularly trisomy 18,13 and Beckwith-Wiedemann syndrome [10].

Ultrasound can diagnose omphalocele and some of its associated malformations as early as 12 weeks of gestation [11, 12]. Congenital heart disease is better evaluated during the second trimester [1].

Most patients are born alive. Survival rates depend on the associated defects, as patients with isolated lesions have a far better prognosis than those with multiple malformations or genetic syndromes [2].

Surgical approach can be done in primary, staged or delayed closure, depending on the size of the defect and co-existing malformations.

The aim of this study is to describe the clinical and epidemiological profile and outcomes of patients born with omphalocele in a reference tertiary care center in Brazil, using data from our center registered in the Latin American Collaborative Study of Congenital Malformations (ECLAMC). Evaluate the prevalence of omphalocele and its association with other congenital defects and genetic diseases, emphasizing the most common cardiac abnormalities and their frequency.

## Methods

This is a retrospective, cross-sectional study using the ECLAMC database, a congenital malformation monitoring program which includes data collected by the Medical Genetics Department of Instituto Fernandes Figueira/FIOCRUZ (IFF/FIOCRUZ). It contains information about live births and stillbirths, the description of birth defects, clinical and obstetric history, results of pregnancy exams, and parental socioeconomic data.

This research follows the norms defined by resolutions 466/12 and 510/16 of the Brazilian Ministry of Health. Informed consent for participation is waived by the Ethics Committee of Instituto Fernandes Figueira through process number 4,673,277.

Data from all children born with the diagnosis of omphalocele between January 1st, 2016 to December 31st, 2019 were reviewed. We analyzed gender, maternal age, parity, birthweight, gestational age at birth, associated malformations, chromosomal defects, and perinatal outcomes. For gestational age at birth, preterm was considered: less than 259 days (37 weeks), term: 259–293 days (37–41 weeks) and post term: 294 days (42 weeks) or longer. Descriptive statistics used frequencies and percentages using the Excel® program for Microsoft 365®.

## Results

During the study period, 4.260 children were born in our unity, 4.064 live births and 196 stillbirths. There were 737 patients with a congenital defect, of which 38 were omphaloceles. Of these, 27 were live births and 11 were stillbirths. One live born patient was excluded for incomplete data. The way of birth of the babies was cesarean section in 27 cases (73%) and spontaneous delivery in 10 (27%).

Twenty-three babies were male (62.2%), 7 were female (18,9%) and 7 were indeterminate (18,9%). Most pregnant women were between 20 and 35 years old (n = 27), none was below 20 and 10 were over 34. Twenty-three women were multiparous (62.2%) and 14 were primiparous (37,8%). Only one case of gestational diabetes was registered. 81% of the babies were appropriate for gestational age and 18 (48.6%) were born weighing 2,500 g or more. Half of the deliveries (51.3%) were preterm (Table 1).

**Table 1:** Characteristics of all patients born with omphalocele

		*n* (%)*
Gender	Male	23 (62.2)
	Female	7 (18.9)
	undetermined	7 (18.9)
Maternal Age	≥ 20 to < 35 years	27 (73.0)
	≥ 35 years	10 (27.0)
	< 20 years	0
Parity	Multiparous	23 (62.2)
	Primiparous	14 (37.8)
Birthweight	≥ 2,500 g	18 (48.6)
	< 2,500 g > 1,500 g	8 (21.6)
	< 1,500 g > 1,000 g	7 (19.9)
	< 1,000 g	4 (10.8)
Appropriateness of weight	SGA¹	3 (8.1)
	AGA²	30 (81.1)
	LGA³	4 (10.8)
Gestational age	Preterm	19 (51.3)
	Term	18 (48.7)
Birth	Live birth	26 (70.3)
	Stillbirth	11 (28.7)

* Related to the total number of patients with omphalocele (37).

¹small for gestational age.

²appropriate for gestational age

³large for gestational age

Only 10.8% (4/37) patients had isolated omphalocele, ten (27.0%) had a genetic condition: 4 Edwards syndrome, 2 Beckwith-Wiedemann syndrome, 2 Donnai-Barrow syndrome, 1 Patau syndrome and one a 15-chromosome monosomy. Complex or malformation sequences were found in 5 cases (13.5%), 4 body stalk and 1 sirenomelia.

The data on the size of the omphalocele was incomplete in 20 of the 37 cases (54.1%), 14 (37.8) were described as large or giant and 3 (8.1%) as small.

Heart disease was the most prevalent association, present in 45.9% of the patients. The distribution of associated anomalies is shown in Table 2.

**Table 2:** Associated anomalies

	*n* (%)*		*n* (%)*
Cardiac	17 (45.9)	Glandular	5 (13.5)
Musculoskeletal	15 (40.5)	Diaphragmatic	2 (5.4)
Central Nervous System	13 (35.1)	Body stalk	4 (10.8)
Craniofacial	10 (27.0)	Gastrointestinal tract	3 (8.1)
Genitourinary	7 (18.9)	Splenic	3 (8,1)
Kidney	5 (13.5)	Sirenomelia	1 (2.7)
Pulmonary (Hypoplasia)	6 (16.2)		

*Related to the total number of patients with omphalocele (37).

Congenital heart disease was present in 17 patients, most of them non-syndromic (10/17). A genetic syndrome was present in 6 cases (35.3%) and a malformation sequence in one (5.9%). Five patients (29.4%) had more than one structural cardiac anomaly. The most prevalent heart defects were tetralogy of Fallot (TOF) and ventricular septal defect (VSD). In five patients a patent foramen oval (PFO) or patent ductus arteriosus (PDA) was present without other heart defects, and were not considered pathological, as echocardiographic evaluation was performed in newborns in the first few days of life. Table 3 shows in detail the congenital heart diseases and their association with syndromes. Patients with isolated PDA or PFO were excluded from analysis.

**Table 3:** Cardiac anomalies associated with omphalocele. Stratified by syndromic or non-syndromic

	Non-syndromic	Syndromic	Unspecified aneuploidy	Malformation sequence	*n* (%) *
**TOF**	3	1	0	0	4 (23.5)
**Coarctation of Aorta**	0	1	0	0	1 (5.9)
**ASD**	1	1	0	0	2 (11.8)
**VSD**	2	0	1	0	3 (17.6)
**VSD + ASD**	0	1	0	0	1 (5.9)
**VSD + PLSVC**	0	1	0	0	1 (5.9)
**Complex Heart Disease**	1	0	0	0	1 (5.9)
**Total AVSD**	0	1	0	0	1 (5.9)
**Acardia**	1	0	0	0	1 (5.9)
**HLHS**	1	0	0	0	1 (5.9)

*Related to the total number of patients with omphalocele (37).

**TOF – tetralogy of Fallot, ASD - atrial septal defect, VSD – ventricular septal defect, AVSD – atrioventricular septal defect, PLSVC -** **Persistent left superior vena cava, HLHS – hypoplastic left heart syndrome.**

Among the 11 stillbirths 54% (6/11) had a genetic syndrome or a malformation complex, 54% (6/11) had pulmonary hypoplasia and 81.8% (9/11) had congenital heart disease. Only one stillbirth had congenital heart disease and a genetic syndrome (Edwards syndrome and atrial septal defect). Ten stillbirths were preterm and 9 weighed less than 1,500 g. Six were male, 4 had undetermined gender and only one was female.

Of the 26 live births, 46.1% (12/26) were discharged from hospital, among them all 4 with isolated omphalocele. One was transferred from neonatal intensive care unit after 3 months of age and we did not analyze long term outcome.

Overall mortality rate was 64.9% (11 stillbirths and 13 live births). All cases of isolated omphalocele survived and were discharged from hospital. All deaths occurred when there was an association with two or more major congenital defects, especially among stillbirths.

## Discussion

Although omphalocele has a low prevalence, compared to other malformations, it has high morbimortality, and when associated with other malformations, there is a high impact on quality of life and outcome [2].

According to SINASC (abbreviation for “Information System on Live Births” in Portuguese), between 2016 and 2019, the prevalence of omphalocele was 0.8:10,000 in Brazil and Rio de Janeiro State. On the other hand, it is as high as 1.7:10,000 in Rio de Janeiro city [13]. This difference is justified for the specialized tertiary care hospitals located in the city. These data in accordance with literature, around 1.0-2.6/10,000 live births [2, 3]. It is suggested that the low prevalence of omphalocele in SINASC can be explained by underreporting of cases and low reliability of records [14].

The number of live births with omphalocele in our institution corresponds to 37.5% of these live births in Rio de Janeiro State, and 46.5% in Rio de Janeiro City [13]. This occurs due to our profile as tertiary care referral hospital for Fetal Medicine, Pediatric Surgery and Medical Genetics, in Rio de Janeiro State.

This study showed a prevalence of 62.2% in male gender (23/37) and a similar number of female and undetermined gender, 18.9% each. Furthermore, male [6] and undetermined gender [4] were present in 90.9% of stillbirths. Male gender prevalence is in accordance with literature, as Marshall et al. [8] in a retrospective cohort study, with 12 million births, estimated a greater risk of omphalocele in males. Regarding maternal age, in most studies, there was no stablished pattern, although there was a higher prevalence of young maternal age [15, 16]. Salihu et al. in a cohort with 2 million births describes a higher prevalence in extreme maternal ages [17]. Our study found 26.3% (10/37) of advanced maternal age (> 35 years old), and no mothers bellow 20 years old.

Literature suggests low birth weight and prematurity are expected in omphalocele newborns [1, 17, 18]. In accordance, low birth weight was present in 21.6% of the patients, very low birth weight in 19.9% and extreme low birth weight in 10.8%, accounting for 51.4% of our sample. Nonetheless, 81.1% of the babies (30/37) had appropriate weight for gestational age and only 8.1% were small for gestational age. There were 51.3% preterm, in agreement with works that show a clear association omphalocele/prematurity in the presence of multiple congenital anomalies with premature birth being the obstetric complication most associated with conditions that threaten life, such as Edwards Syndrome [8, 19]. In the same period, in Rio de Janeiro City, the overall rate of prematurity was 10.9% [13].

Isolated omphalocele was present in only 4 patients (10.5%), although literature says it is rare, our sample shows a much lower number of cases than most series, especially those with largest samples [2, 8, 20]. Genetic conditions were present in 10 patients and Edwards syndrome was the most prevalent. The same result was found in important cohorts as Marshall et al [8] and Calzolari et al [21]. Beckwith-Wiedemann, Donnai-Barrow and Patau syndrome were also present, and one patient had a 15-chromosome deletion. Although described as common, no Down syndrome was found in this cohort [4, 8].

Cardiac and musculoskeletal were the most prevalent associated malformations, followed by central nervous system, craniofacial and genitourinary anomalies. Involvement of organs and systems is shown in Table 2. The association of multiple malformations is common and is associated to prognosis [4].

Congenital heart disease was present in 45.9% (17/37). The most frequent were VSD in 29.4% (5/17) and TOF in 23.5% (4/17). Although present and described in literature as common, PDA and PFO were not considered congenital malformations because echocardiograms performed in the first week of life [8]. The association of congenital heart disease and omphalocele has been extensively described. Ventricular septal defects, atrial septal defects, tetralogy of Fallot and tricuspid defects are among the most common and their presence is associated to worse outcomes [4, 8].

In this study we found a mortality rate of 64.9%, which is higher than literature. This is due to the great number of associated anomalies found in these patients. It is possible that the size of the omphalocele also contributed to the poor prognosis, however it is not possible to analyze this data due to the absence of this information in 54.1% of the records of ECLAMC database. It is important to consider that most of these papers come from countries where termination of pregnancy is permitted. This is especially important in cases which omphalocele is associated to major congenital defects. Termination of pregnancy in cases of congenital abnormalities is forbidden in Brazil, except for acrania. In some studies termination rates can reach 63% [22–25]. In accordance with literature, our cohort showed that isolated omphalocele has a good prognosis, as we had 100% survival rate (4/4) and hospital discharge when no other congenital defect was present [2]. However, the importance of follow-up of babies with omphalocele should be highlighted in order to reduce complications in the medium and long term, such as the need for new surgical procedures, evaluation of nutritional, developmental and aesthetic problems, and enabling the scheduling of medical care and suitable rehabilitation [26].

## Conclusion

Our data showed a good correspondence with the existing literature, most patients with omphalocele were born with other malformations, especially congenital heart disease. Although our mortality rate was higher than literature, different from other countries, Brazilian law does not allow elective termination of pregnancy. The occurrence of concurrent birth defects showed a huge impact on prognosis, since, even if most survive birth, few remained alive and received hospital discharge. Based on these data, fetal medicine and neonatal teams must be able to adjust parents counseling about fetal and neonatal risks, especially when other congenital diseases are present.

## Data Availability

The datasets generated and/or analyzed during the current study are not publicly available due they were taken from ECLAMC Database, but are available from the corresponding author on reasonable request.

## List of Abbreviations

AGA: Adequate for Gestational Age
ASD: Atrial Septal Defect
AVSD: Atrioventricular Septal Defect
ECLAMC: Latin-American Collaborative Study of Congenital Malformations
HLHS: Hypoplastic Left Heart Syndrome
IFF/FIOCRUZ: Instituto Fernandes Figueira/FIOCRUZ
LGA: Large for Gestational Age
PFO: Patent Foramen Oval
PLSVC: Persistent Left Superior Vena Cava
PDA: Patent Ductus Arteriosus
SGA: Small for Gestational Age
SINASC: Information System on Live Births
TOF: Tetralogy of Fallot
VSD: Ventricular Septal Defect
